# The Effect of Knowledge Levels of Breastfeeding Mothers About the Disease on Their Levels of Anxiety During the COVID-19 Pandemic Process

**DOI:** 10.3389/fpubh.2022.856228

**Published:** 2022-07-11

**Authors:** Selda Ayşe Tekiner, Nisa Eda Çullas Ilarslan, Fatih Günay, Gülsen Ayşe Ceyhun Peker

**Affiliations:** ^1^Department of Family Medicine, Ankara University School of Medicine, Ankara, Turkey; ^2^Department of Pediatrics, Ankara Memorial Hospital, Ankara, Turkey; ^3^Department of Pediatrics, Ankara University School of Medicine, Ankara, Turkey

**Keywords:** breast milk, COVID-19, infant, anxiety, pandemic

## Abstract

**Background:**

The health impact of severe acute respiratory syndrome-coronavirus-2 (SARS-CoV-2) spans across all age groups including mothers and their newly born infants; and breastfeeding women during this pandemic deserves special attention due to its short and long-term health implications. When planning the current study, our hypothesis was that the anxiety of transmitting the COVID-19 disease to the baby through breastfeeding would increase among breastfeeding women and it would predominantly be state anxiety. The current study aims to investigate the relationship between the knowledge levels of breastfeeding mothers about COVID-19 and their anxiety levels.

**Materials and Methods:**

This is a cross-sectional study and we aimed to reach all mothers with babies between 0 and 24 months of age who applied to the Healthy Child Care Policlinic of Ankara University Faculty of Medicine Hospital between July 1 and August 31, 2020 Questionnaires measuring the level of knowledge about COVID-19 disease and breast milk and questionnaires measuring anxiety levels were administered to mothers. The state-trait anxiety levels and knowledge levels of breastfeeding mothers about COVID-19 disease, as well as their knowledge levels about breast milk and sociodemographic characteristics were compared.

**Results:**

A total of 145 breastfeeding mothers were included in the study. The trait anxiety was found to be either absent or mild in 89 (61.4%), moderate in 28 (19.3%), and advanced in 28 (19.3%) mothers. The state anxiety level, however, was found to be either absent or mild in 51 (35.2%), moderate in 42 (29%), and advanced in 52 (35.9%) mothers. When mothers' trait and state anxiety levels and their knowledge levels about breast milk, their knowledge levels about COVID-19 disease and their sociodemographic characteristics were compared, it was determined that mothers with 0–6 months old infants had lower state anxiety levels compared to mothers with older infants. However, the anxiety levels of mothers whose children were >12 months old were mostly moderate (*p* < 0.05). There was no statistically significant difference in terms of other variables (*p* < 0.05). A positive correlation was found between the child age and state anxiety level (*p* = 0.027).

**Conclusion:**

Moderate and advanced level of state anxiety was found to be higher than level of trait anxiety parallel to our hypothesis. Among breastfeeding mothers, those with the lowest state anxiety scores were those who had babies between 0 and 6 months. It is important to support breastfeeding mothers in promoting breastfeeding, especially after 6 months of birth. Policymakers, obstetricians and especially the family physicians should be aware that adverse life events may put a higher burden on the emotional wellbeing of breastfeeding women especially after 6 months of birth of the babies. So, breastfeeding promotion, protection and support strategies should be reconsidered specially after 6 months.

## Introduction

COVID-19, initially named 2019-nCoV by the World Health Organization (WHO), was later renamed SARS-CoV-2 by the International Committee on Taxonomy of Viruses and subsequently characterized as a pandemic on March 11, 2020. In Turkey, the first official case diagnosed with COVID-19 was announced on 11 March 2020 and more than 500 million people were infected globally up to that point (1). The health impact of severe acute respiratory syndrome-coronavirus-2 (SARS-CoV-2) spans across all age groups including mothers and their newly born infants; and breastfeeding women during this pandemic deserves special attention due to its short and long-term health implications (2). The evidence about the benefits and advantages of breast milk at all stages of life is well established. In addition to the benefits of breastfeeding on the mother and the baby, it is also known that it has benefits on the health system and health expenditures that burden the society (3). When we searched the literature, whether SARS-CoV-2 can be shed into human milk and transmitted to a child via breastfeeding we found in a systematic review that the authors were not sure whether SARS-CoV-2 transmission via breast milk is possible as of the certainty of the evidence has been assessed very low (4). In another case series study conducted from a different center in Turkey, the authors state that; although SARS-CoV-2 may be excreted into breast milk, this does not mean that the virus will show its viability and/or that breast milk may be a source of infection for infants (5). World Health Organization, United Nations Children's Fund and Royal College of Obstetricians and Gynecologists recommend that breastfeeding should be continued, except in cases where the mother's health is poor due to COVID-19 or the infant needs treatment as a result of COVID-19 infection (6). According to the “Interim Guidance on Breastfeeding and Breast Milk Feeds in the Context of COVID-19” of CDC; people without suspected or confirmed COVID-19 and who have not been in close contact with someone who has COVID-19, do not need to take special precautions when feeding at the breast or expressing milk (7). Despite all this information, there have been recommendations against breastfeeding in the early stages of the COVID-19 pandemic. For example, some governments and health care systems recommended that infected mothers be separated from their infants after birth to reduce the risk of infant COVID-19 infection (8). Spread of unrealistic or erroneous information about the COVID-19 disease can cause panic and fear in societies and even make it difficult to fight the disease. Today, reaching the news is realized through traditional media such as television and radio, as well as social media and offline information flow in the real world, and this situation can play a very important role in the spread of inaccurate information (9). In Turkey in the early period of the pandemic, internet journalism and social media have been the principal sources of information in relation to COVID-19 (10). There may be a possibility that some information received from these sources may be inaccurate and/or misunderstood and concerns around transmission of the infection to their babies may potentially have a significant impact on breastfeeding mothers. Concerns about contamination may increase during breastfeeding, which can lead to increased anxiety levels (11). Anxiety is defined as an organic response, characterized by apprehension and increased surveillance in situations of uncertain danger or potential threats to the integrity of the organism. By now, studies show that there are two complementary concepts of anxiety; a psychophysiological state: state anxiety and a personality trait: trait anxiety. State anxiety reflects the psychological and physiological transient reactions directly related to adverse situations in a specific moment. In contrast, the term trait anxiety refers to a trait of personality, describing individual differences related to a tendency to present state anxiety. Trait anxiety is, therefore, relatively stable over time and considered an important characteristic of patients with anxiety disorders, as they present higher trait anxiety in comparison to healthy individuals (12). When planning the current study, our hypothesis was that the anxiety of transmitting the COVID-19 disease to the baby through breastfeeding would increase among breastfeeding women and it would predominantly be state anxiety.

We aimed to distinguish the anxiety as state or trait anxiety *via* “The State-Trait Anxiety Inventory” (STAI). STAI is a self-report questionnaire composed of 40 items divided into two equal 20-item parts; the participant indicates how he/she feels generally by rating himself on a 4-point Likert scale ranging from: almost never (1) to almost always (3). Low scores indicate calmness and confidence, while high scores reflect high anxiety, stress and worry levels (13).

Besides, we aimed to investigate the relationship between anxiety and the level of knowledge about COVID-19 disease, the level of knowledge with respect to breastfeeding and the sociodemographic characteristics of breastfeeding mothers.

## Materials and Methods

The current research was planned as a cross-sectional study between July 1 and August 31, 2020. We planned to include mothers having a baby who are breastfeeding age. We aimed to reach all mothers with babies between 0 and 24 months of age who applied to the Healthy Child Care Policlinic of Ankara University Faculty of Medicine Hospital. Between July 1 and August 31 2020, a total of 600 applications were received to the specified polyclinic. The researcher offered the women to participate to the research and distributed the written informed consent form. The study was carried out on those who volunteered. First of all the three-stage questionnaire form was administered to five women to be pretested. The questionnaire was filled out face to face in a quiet area. It took ~15 min to complete the form. There was no problem in the pre-testing application and then were applied to all volunteers. During the study period, 280 women were informed about the study. Mothers who are not breastfeeding and/or with a diagnosed psychiatric illness were excluded. Of the women 135 were excluded; because 70 women had stopped breastfeeding before the pandemic, 3 had a psychiatric illness, 21 refused to participate in the study, and 41 did not complete the questionnaire. So, this current study included 145 subjects with a compliance rate of 51.70%. The waiting times of the mothers and babies in the outpatient clinics were very short, both due to hospital rules and because of the mothers' fear of pandemics. Therefore, the number of volunteers who agreed to participate in the study was limited.

The study was approved by the Clinical Research Ethics Committee of the Faculty of Medicine of the Ankara University with a reference number of i6-384-20, dated 13.07.2020.

Sociodemographic characteristics of the mothers who were included in the study, including age, educational status, number of individuals living in the family, number of pregnancies, number of children, number of breastfed children, and monthly income of the family were recorded. In addition, mothers were also asked if they have ever got COVID-19. A three-stage questionnaire form was administered in person to the mothers included in the study by the principal researcher, who carried out the study in a quiet area via face-to-face interviews. In the first stage, a questionnaire measuring the knowledge levels of mothers about COVID-19 disease was administered, the second stage involved a questionnaire evaluating the level of knowledge about breast milk and breastfeeding attitudes, and in the third stage, “The State and Trait Anxiety Inventory” was applied to measure the anxiety levels of mothers.

The first stage questionnaire covered some 36 questions about the symptoms of COVID-19, transmission routes, the course of the disease during pregnancy and breastfeeding in order to measure the knowledge levels of the mothers in relation to COVID-19. The answers to these questions were categorized as either “true or false”. Mothers' knowledge about the content, benefits, and necessity of breast milk as well as breastfeeding during the pandemic were covered by 13 statements to evaluate the knowledge levels of mothers about breast milk and their attitudes to breastfeeding at the second stage. The answers to these statements were also categorized as either “true or false”. A similar scoring system was used in both questionnaires. Answers to the questions in each survey were scored out of 100.

A questionnaire by Spielberger (14), adapted to Turkish society by Öner and Le Compte and consisting of 20 questions was applied to measure the state and trait anxiety levels of mothers (15). The state anxiety scale determines the permanence of interpreting the amount and severity of the threat perceived by the individual at a given moment, while the trait anxiety scale determines the individual's susceptibility to anxiety regardless of the situation she is in and the conditions she experiences. Questions in both scales include a four-point Likert scale ranging from “Not at all” to “Completely”. The total score obtained from both scales has a minimum score of 20 points and a maximum of 80 points. Anxiety scores are classified as “no or low anxiety” (20–37), “moderate anxiety” (38–44), and “high anxiety” (45–80).

The mothers' trait and state anxiety levels and their knowledge about COVID-19 disease, their knowledge about breast milk, and their sociodemographic characteristics were compared.

### Statistical Analysis

The SPSS 25.0 (IBM Corporation, Armonk, New York, United States) program was used in the analysis of the variables. The conformity of the data to the normal distribution was evaluated with the Shapiro-Wilk Francia test, while the homogeneity of variance was evaluated with the Levene test. According to the quantitative data, the Mann-Whitney U test was used together with the Monte Carlo results to compare two independent groups with each other. In the comparison of more than two groups according to the quantitative data, the Jonckheere-Terpstra test was used with the Monte Carlo results, while the Dunn's test was used for the *post-hoc* analysis. The Spearman's rho test was used to examine the correlation of the variables with each other. In the comparison of categorical variables with each other, the Pearson Chi-Square Fisher-Freeman-Holton tests were used with the Monte Carlo Simulation technique, and the column ratios were compared with each other and expressed according to the Benjamini-Hochberg corrected *p*-value results. While quantitative variables were expressed as mean (standard deviation) in the tables, categorical variables were shown as *n* (%). The variables were analyzed at 95% confidence level, and a *p*-value of < 0.05 was considered to be significant.

## Results

A total of 145 mothers were included in the study. The mean age of the mothers was 28.9 ± 6.0 years. With respect to education level, 19 (13.1%) had attended primary school, 22 (15.2%) secondary school, 60 (41.4%) high school, and 44 (30.3%) university. Of the mothers, 59 (40.7%) had one child, 65 (44.8%) had two, and 21 (14.5%) had three or more children. While 130 (89.7%) of the mothers included in the study lived within a nuclear family, 15 (10.3%) lived with other family members. Of the breastfed infants, 95 (65.5%) were 0–6 months, 36 (24.8%) 6–12 months, and 14 (9.7%) >12 months old. While 52 (35.9%) of the study participants lived in a house of three individuals, 62 (42.8%) lived in a house of 4, and 31 (21.3%) in a house of 5 or more persons. None of the mothers had got COVID-19 till now.

In the second stage, women's attitudes about breastfeeding were evaluated and the instructions were asked to be answered as “yes” or “no”. All of the participants answered “no” to the instruction “I would stop breastfeeding if I were having COVID-19”.

Trait anxiety levels were found to be absent or mild in 89 (61.4%), moderate in 28 (19.3%), and advanced in 28 (19.3%) of the mothers. The level of state anxiety was found to be absent or mild in 51 (35.2%), moderate in 42 (29%), and advanced in 52 (35.9%) mothers (Table 1).

**Table 1:** General characteristics of 145 mothers included in the study.

Maternal age (years)^*^	**28.9 ± 6.0**
**Mothers' education level**, ***n*** **(%)**	
Primary school	19 (13.1)
Middle school	22 (15.2)
High school	60 (41.4)
University	44 (30.3)
**Number of children**, ***n*** **(%)**	
1	59 (40.7)
2	65 (44.8)
≥3	21 (14.5)
**Breastfed child's age (months)**, ***n*** **(%)**	
0–6 months	95 (65.5)
6–12 months	36 (24.8)
>12 months	14 (9.7)
**Does she live in a nuclear family?** ***n*** **(%)**	
No	15 (10.3)
Yes	130 (89.7)
**Number of individuals living in the same house** ***n*** **%**	
3	52 (35.9)
4	62 (42.8)
≥5	31 (21.3)
**Monthly income (Turkish lira)**, ***n*** **(%)**	
1,000–2,999	33 (22.8)
3,000–4,999	53 (36.6)
5,000–5,999	46 (31.7)
>6,000	13 (9.0)
Level of knowledge about breast milk^*^	86.5 ± 11.1
Level of knowledge about Covid-19^*^	80.9 ± 9.4
Trait anxiety level^*^	33.9 ± 12.8
State anxiety level^*^	40.9± 9.0
**Trait anxiety level**, ***n*** **(%)**
None or mild anxiety	89 (61.4)
Moderate anxiety	28 (19.3)
Advanced anxiety	28 (19.3)
**State anxiety level**, ***n*** **(%)**	
None or mild anxiety	51 (35.2)
Moderate anxiety	42 (29.0)
Advanced anxiety	52(35.9)

* *Mean ± Standard Deviation*.

**Figure 1:**
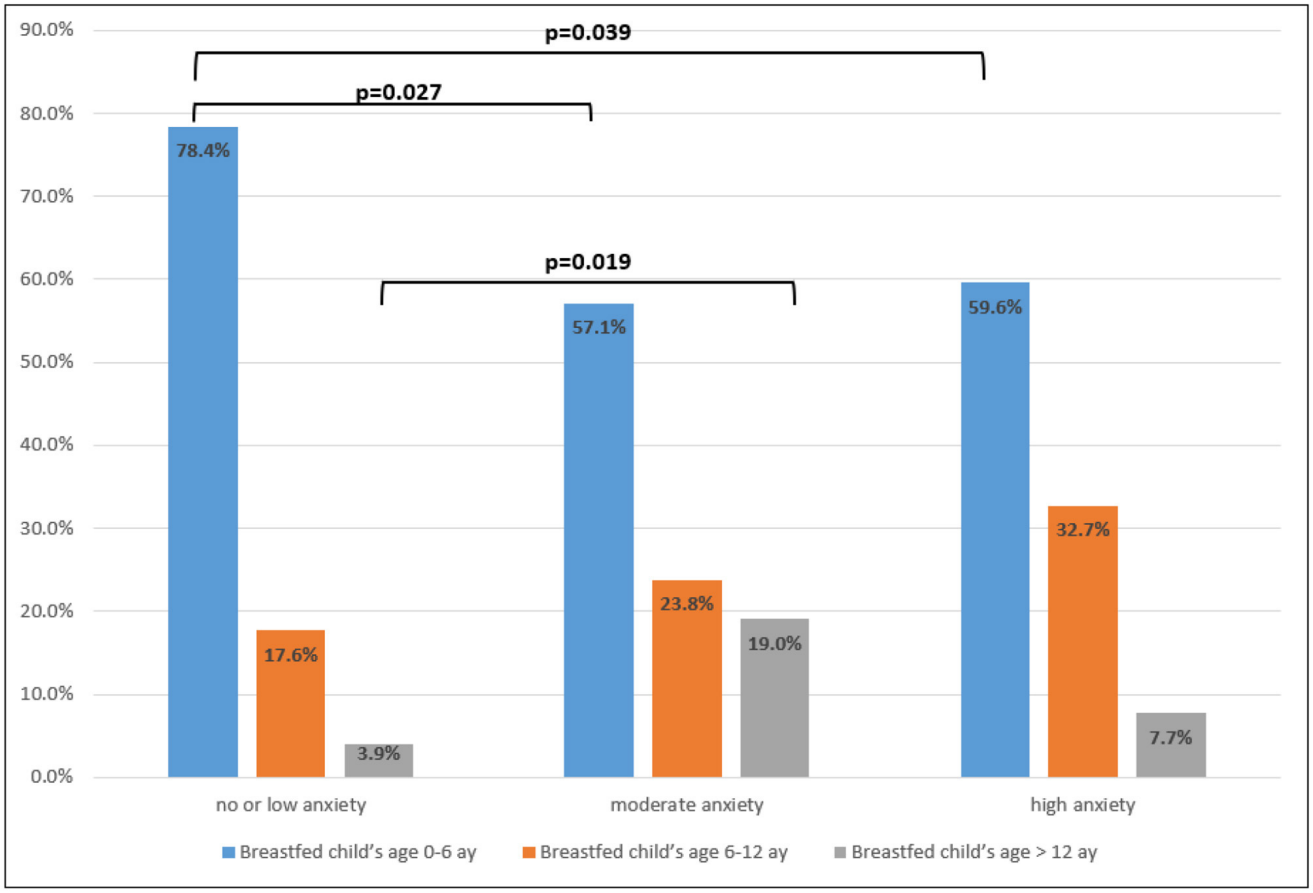
The state anxiety level of mothers who breastfed infants younger than 6 months tended to be mild and the state anxiety level of mothers who breastfed infants older than 12 months tended to be moderate (*p* < 0.05).

When the relationship between trait and state anxiety levels and maternal age, mother's education level, whether she lived in a nuclear family or not, number of living children, age of breastfed baby, monthly income, level of knowledge about breast milk and level of knowledge about COVID-19 disease were analyzed, it was determined that the state anxiety level of mothers with 0–6 month-old infants was much milder than that in mothers with older infants, while the state anxiety level of mothers with children > 12 months was more moderate (*p* < 0.05). There was no statistically significant difference between other variables (*p* > 0.05) (Table 2; Figure 1).

**Table 2:** The relationship between mothers' anxiety levels and their knowledge levels and sociodemographic characteristics.

	**Trait anxiety level**			**State anxiety level**		
	**No or low anxiety**	**Moderate anxiety**	**High anxiety**	**No or low anxiety**	**Moderate anxiety**	**High anxiety**
	***n*** **(%)**	***n*** **(%)**	***n*** **(%)**	***n*** **(%)**	***n*** **(%)**	***n*** **(%)**
**Age (year)**						
≤30	53 (59.6)	17 (60.7)	15 (53.6)	36 (70.6)	22 (52.4)	27 (51.9)
>30	36 (40.4)	11 (39.3)	13 (46.4)	15 (29.4)	20 (47.6)	25 (48.1)
*p*-value		0.884^c^			0.095^c^
**Mother's education level**						
Primary school	11 (12.4)	4 (14.3)	4 (14.3)	8 (15.7)	5 (11.9)	6 (11.5)
Middle school	12 (13.5)	5 (17.9)	5 (17.9)	9 (17.6)	5 (11.9)	8 (15.4)
High school	38 (42.7)	11 (39.3)	11 (39.3)	17 (33.3)	21 (50.0)	22 (42.3)
University	28 (31.5)	8 (28.6)	8 (28.6)	17 (33.3)	11 (26.2)	16 (30.8)
*p*-value		0.986^f^			0.833^c^
**Nuclear family**						
No	9 (10.1)	3 (10.7)	3 (10.7)	6 (11.8)	4 (9.5)	5 (9.6)
Yes	80 (89.9)	25 (89.3)	25 (89.3)	45 (88.2)	38 (90.5)	47 (90.4)
*p*-value		0.999^f^			0.946^f^
**Number of living children**						
1	37 (41.6)	13 (46.4)	9 (32.1)	26 (51.0)	11 (26.2)	22 (42.3)
>1	52 (58.4)	15 (53.6)	19 (67.9)	25 (49.0)	31 (73.8)	30 (57.7)
*p*-value		0.525 ^c^			0.053 ^c^
**Breastfed child's age (months)**						
0–6	60 (67.4)	18 (64.3)	17 (60.7)	40 (78.4)^B^^C^	24 (57.1)	31 (59.6)
6–12	18 (20.2)	9 (32.1)	9 (32.1)	9 (17.6)	10 (23.8)	17 (32.7)
>12	11 (12.4)	1 (3.6)	2 (7.1)	2 (3.9)	8 (19.0)^A^	4 (7.7)
*p*-value		0.432^f^			**0.047^f^**
**Monthly income (Turkish lira)**						
1,000–2,999	19 (21.3)	5 (17.9)	9 (32.1)	12 (23.5)	7 (16.7)	14 (26.9)
3,000–4,999	32 (36.0)	13 (46.4)	8 (28.6)	22 (43.1)	15 (35.7)	16 (30.8)
5,000–5,999	29 (32.6)	9 (32.1)	8 (28.6)	13 (25.5)	17 (40.5)	16 (30.8)
>6,000 tl	9 (10.1)	1 (3.6)	3 (10.7)	4 (7.8)	3 (7.1)	6 (11.5)
*p*-value		0.719^f^			0.628^f^
	**Median (q1/q3)**	**Median (q1/q3)**	**Median (q1/q3)**	**Median (q1/q3)**	**Median (q1/q3)**	**Median (q1/q3)**
**Breast milk knowledge level**	84.6 (76.9/100)	92.3 (84.6/92.3)	84.6 (84.6/92.3)	84.6 (76.9/92.3)	92.3 (84.6/100)	84.6 (76.9/92.3)
		0.664^j^			0.719^j^
**COVID-19 knowledge level**	80.6 (75.0/88.9)	80.6 (76.4/87.5)	80.6 (76.4/88.9)	83.3 (77.8/88.9)	81.9 (75.0/88.9)	80.6 (73.6/88.9)
		0.780^j^			0.208^j^

*u Mann-Whitney U-Test (Monte Carlo)*.

f *Fisher Freeman Halton (Monte Carlo); post-hoc Test: Benjamini-Hochberg correction*.

c *Pearson Chi Square Test (Monte Carlo)*.

j *Jonckheere-Terpstra Test (Monte Carlo); post-hoc Test: Dunn's Test. q1: 25 percentile. q3: 75 Percentile*.

A *Significant to no or low anxiety group*.

B *Significant to moderate anxiety group*.

C *Significant to high anxiety group*.

There was a positive correlation between infant age and state anxiety level in the correlation analysis between anxiety level and mother's age, mother's education level, number of pregnancies, number of living children, age of breastfed infant and monthly income (*p* = 0.027) (Table 3).

**Table 3:** Correlation analysis between anxiety level and sociodemographic characteristics.

	**Trait anxiety level**	**State anxiety level**
**Maternal age**		
R	0.021	0.155
P	0.804	0.062
**Mother education level**		
R	0.046	0.008
P	0.579	0.927
**Number of individuals living in the same house**		
R	0.081	0.110
P	0.336	0.190
**Number of pregnancies**		
R	0.011	0.130
P	0.893	0.118
**Number of children**		
R	0.009	0.124
P	0.916	0.137
**Breastfed child's age**		
R	0.050	0.183
P	0.553	**0.027**
**Monthly income**		
R	0.001	0.049
P	0.992	0.561

*Spearman's rho test. r: Correlation Coefficient*.

## Discussion

Our study was carried out shortly after the COVID-19 disease was declared as pandemic by the World Health Organization. Nevertheless, breastfeeding mothers' state anxiety level was mild and there was a positive correlation between the level of state anxiety and the age of the infant. We can attribute this to the positive effect of education and support on breastfeeding given during pregnancy and childbirth in our country and that this effect may be decreasing over time.

It was determined that the level of knowledge about breast milk, the level of knowledge about COVID-19 disease and sociodemographic characteristics were not effective on the breastfeeding behavior. In Costantini's study from United Kingdom which is very similar to ours, emotional states of breastfeeding mothers were evaluated during COVID-19 pandemic via Generalized Anxiety Disorder Questionnaire and found that mothers with more than one child showed higher negative emotional states, especially anxiety symptoms (16). We used state and trait anxiety questionnaire and aimed to understand whether state anxiety status is associated with pandemic. In our study the same researcher performed the survey face-to-face by following social distancing rules very strictly and these are strong features of this study. All of our participants and 94% of the participants in the Costantini's study answered “yes” to the question “do you continue breastfeeding even if you show symptoms of COVID-19”. Participants in the same study who breastfed for a longer period and those with more than one child were more in favor of the use of human milk donation and wet nursing in case of COVID-19 symptoms. While human milk donation and wet nursing were stated in our study, no woman indicated that she would prefer them. This difference can be attributed to the availability of Human Milk Banks in the UK and the fact that this has been customary since before. According to the WHO if a mother has COVID-19 and is not able to breastfeed her child, expressed human milk, as well as donor human milk and wet nursing, should be advised by health professionals (17). However, this recommendation does not seem to be used practically in our study. Findings in Costantini's and our studies show that the age of the breastfed child and having more than one child increase the anxiety in mothers, especially during the pandemic period (16).^1^,^2^

Unlike this and our study, it has been shown in a study conducted in Mexico in the early period of the pandemic that 64.0% of women living in households with children under 3 years of age believe that, mothers with COVID-19 should not breastfeed, and 19.8% do not know whether mothers should breastfeed or not. The reason was declared as “because the virüs is transmitted by milk”. And this belief was more prevalent among socio-economically disadvantaged families, which could be at higher risk of not initiating or continuing breastfeeding during the pandemic. In this study the authors stated that the Mexican government endorsed the recommendation on breastfeeding during the COVID-19 pandemic; but communication was sporadic, inconstant and unequal across types of media, and there was a notion that mothers with COVID-19 should not breastfeed. The authors showed that during the pandemic period, most of the information about breastfeeding is obtained from the internet, newspapers and television, and 31% of this information is neutral and 11% is negative. Similar to our study, beliefs about breastfeeding during the COVID-19 pandemic did not differ according to age (8). We can attribute the determination and consistency of the women in our study to breastfeeding, regardless of the pandemic, to the information and campaigns that have been made about the benefits of breast milk so far.

It has been reported that psychological problems such as anxiety and depression increased in society due to social isolation, during the COVID-19 pandemic. Studies have reported that this process had both positive and negative effects on the breastfeeding behavior. As an example of positive effect, Ceulemans' study showed that 81% of women who declared that they changed their breastfeeding habits during the pandemic, stated that they breastfed their babies more frequently than in the pre-pandemic period (18). The main reasons for this increase were that staying home with the infant (as a result of the lockdown) facilitated breastfeeding, and the woman's desire to protect her infant against coronavirus through breastmilk. Similarly, in the study of Brown from the United Kingdom, some women stated that their breastfeeding experiences were positively affected due to the increase in the time spent at home, less pressure and fewer visitors (19). In the study of Ceulemans, the main reasons for a decline in breastfeeding reported by the women were a reduction in milk production due to concerns about the virus, increased care giving responsibilities, the need to work from home and the combination with other childcare responsibilities at home (20). Again in this study, the author stated that almost half of the women experienced depressive or anxious symptoms during the COVID-19 lockdown in Belgium and of the breastfeeding women 4.7% had severe anxiety disorder. Twelve percentage of women who have stopped breastfeeding attributed this to staying at home due to COVID-19, heavy workload at home, working from home, and most importantly, reduced milk production due to COVID anxiety. The women in our study did not mention that they experienced reduced milk production due to stress or the difficulty of breastfeeding with excessive workload and there was no woman who stopped or thought to stop breastfeeding during this period. Ceulemans' and Brown's studies were also done in the early period of the pandemic, but unlike them, our study was carried out face to face and this is the feature that makes it different and superior.

Another indirect positive effect of the pandemic has been reported that children became ill less frequently during this period, as breastfeeding mothers were able to spend more time taking care of their children at home (21). On the other hand, it has been reported that during this period, mothers were also concerned that both they and their babies would become infected (22, 23). In a study by Brown and Shenker, it was reported that 13.2% of breastfeeding mothers had concerns about the safety of breastfeeding during the pandemic (19).

In Ceulemans' study, the prevalence of major depression was found to be approximately 14% among breastfeeding mothers during the pandemic (18). In a similar study by Sakalidis et al., it was reported that 24.5% of breastfeeding mothers developed anxiety and that more than half of them experienced at least moderate anxiety during the pandemic (24). Similar to the studies in the literature, the current study found that 38.6% of the mothers had moderate or severe trait anxiety, and 64.8% had moderate or severe state anxiety.

In Brown's study, it was reported that mothers who stopped breastfeeding had received information about the risks of breastfeeding primarily from their healthcare professionals, friends or family. It has been reported that the main reason mothers ceased breastfeeding was due to their inability to receive adequate professional support (19). In a study by Wu et al., it was reported that the anxiety level of mothers with a high level of education was higher during the pandemic period (25). In the current study, no relationship was found between anxiety level and education level. In addition, no relationship was found between the level of knowledge about breast milk and the level of anxiety.

In a study by Fry et al., it was reported that mothers' attitudes and behaviors regarding breastfeeding during the pandemic were not related to income level, number of pregnancies, maternal age and ethnicity. In the study, it was reported that 91% of mothers with high socioeconomic status did not change their breastfeeding behavior during the pandemic (26). In the current study, it was shown that the state anxiety level of mothers with infants younger than 6 months was mild compared to that of breastfeeding mothers with older infants. There was a positive correlation between the level of state anxiety and the age of the breastfed infant. Similar to the studies in the literature, no relationship was found between anxiety level and maternal age, number of pregnancies, number of children, and income level.

Although our study is the first study conducted in our country using STAI during the COVID-19 pandemic, it has some limitations. The first is that only breastfeeding mothers were included in the study, and the sample size was limited. On the other hand the strength of our study was obtaining the relevant data from the mothers via face to face interviews during the early period of the pandemic.

As a result, most of the mothers experienced anxiety about breastfeeding regardless of their sociodemographic characteristics, levels of knowledge about breast milk and COVID-19 during the early stages of pandemic. And especially moderate and advanced level of state anxiety was found to be higher than level of trait anxiety parallel to our hypothesis. Among breastfeeding mothers, those with the lowest state anxiety scores were those who had babies between 0 and 6 months. It is important to support breastfeeding mothers in promoting breastfeeding, especially after 6 months of birth. Policymakers, obstetricians and especially the family physicians should be aware that adverse life events may put a higher burden on the emotional wellbeing of breastfeeding women especially after 6 months of birth of the babies. So, breastfeeding promotion, protection and support strategies should be reconsidered specially after 6 months.

## Data Availability Statement

The original contributions presented in the study are included in the article/supplementary material, further inquiries can be directed to the corresponding author.

## Ethics Statement

Written informed consent was obtained from the individual(s) for the publication of any potentially identifiable images or data included in this article.

## Author Contributions

ST, NÇ, and FG contributed to conception and design of the study. GC organized the database. ST performed the statistical analysis and wrote the first draft of the manuscript. NÇ, FG, and GC wrote sections of the manuscript. All authors contributed to manuscript revision, read, and approved the submitted version.

## Conflict of Interest

The authors declare that the research was conducted in the absence of any commercial or financial relationships that could be construed as a potential conflict of interest.

## Publisher's Note

All claims expressed in this article are solely those of the authors and do not necessarily represent those of their affiliated organizations, or those of the publisher, the editors and the reviewers. Any product that may be evaluated in this article, or claim that may be made by its manufacturer, is not guaranteed or endorsed by the publisher.

## Footnotes

^1^https://covid19.who.int/ (accessed April 30, 2022).

^2^https://www.who.int/news-room/commentaries/detail/breastfeeding-and-covid-19 (accessed April 30, 2022).
